# Occurrence of the *mcr-1* Colistin Resistance Gene and other Clinically Relevant Antibiotic Resistance Genes in Microbial Populations at Different Municipal Wastewater Treatment Plants in Germany

**DOI:** 10.3389/fmicb.2017.01282

**Published:** 2017-07-11

**Authors:** Norman Hembach, Ferdinand Schmid, Johannes Alexander, Christian Hiller, Eike T. Rogall, Thomas Schwartz

**Affiliations:** ^1^Bioengineering and Biosystems Department, Karlsruhe Institute of Technology, Institute of Functional Interfaces Eggenstein-Leopoldshafen, Germany; ^2^Zweckverband Kläranlage Steinhäule Neu-Ulm, Germany

**Keywords:** colistin resistance, antibiotic resistance genes, opportunistic bacteria, wastewater treatment, qPCR

## Abstract

Seven wastewater treatment plants (WWTPs) with different population equivalents and catchment areas were screened for the prevalence of the colistin resistance gene *mcr-1* mediating resistance against last resort antibiotic polymyxin E. The abundance of the plasmid-associated *mcr-1* gene in total microbial populations during water treatment processes was quantitatively analyzed by qPCR analyses. The presence of the colistin resistance gene was documented for all of the influent wastewater samples of the seven WWTPs. In some cases the *mcr-1* resistance gene was also detected in effluent samples of the WWTPs after conventional treatment reaching the aquatic environment. In addition to the occurrence of *mcr-1* gene, *CTX-M-32, blaTEM, CTX-M, tetM, CMY-2*, and *ermB* genes coding for clinically relevant antibiotic resistances were quantified in higher abundances in all WWTPs effluents. In parallel, the abundances of *Acinetobacter baumannii, Klebsiella pneumoniae*, and *Escherichia coli* were quantified via qPCR using specific taxonomic gene markers which were detected in all influent and effluent wastewaters in significant densities. Hence, opportunistic pathogens and clinically relevant antibiotic resistance genes in wastewaters of the analyzed WWTPs bear a risk of dissemination to the aquatic environment. Since many of the antibiotic resistance gene are associated with mobile genetic elements horizontal gene transfer during wastewater treatment can't be excluded.

## Introduction

Antibiotic-resistant intestinal bacteria enter the environment through sewage water treatment plants. Some survive or even multiply during the waste water treatment and are capable of transferring genes to other microorganisms (Davies et al., 2006; Rizzo et al., 2013; Berendonk et al., 2015). There is a potential risk of people getting colonized with these bacteria, for example via contact with wastewater contaminated surface water (Ferrer et al., 2012). The resistant bacteria can cause infections which are difficult to treat because of the insensitivity to antibiotics. It is therefore in the interest of our society to quickly determine whether and how resistant bacteria spread via sewage water—and how this could be prevented.

The worldwide increase of antibiotic-resistant bacteria is considered as a major challenge by the World Health Organization (WHO, 2014), the US Centers for Disease Control and Prevention, and the German Antibiotic Resistance Strategy (DART, 2020; The Federal Ministry of Health, 2012). To minimize the leaking of antibiotics or antibiotic-resistant bacteria into the environment, the use of antibiotics in human and veterinary medicine needs to be reduced. Indications of the importance of sewage water and wastewater treatment plants (WWTP) for the spread of antibiotic-resistant pathogens were already described (Rizzo et al., 2013; Alexander et al., 2015, 2016).

A particular danger is represented by pathogens with a resistance against last resort antibiotics. It can be very difficult to cure patients that suffer from an infection with this kind of resistant bacteria. These serious concerns have been catalyzed by the rapid increase in carbapenemase-producing *Enterobacteriaceae* expressing enzymes such as KPC-2 (*Klebsiella pneumoniae* carbapenemase-2) and NDM-1 (New Dehli metallo-beta-lactamase; Kumarasamy et al., 2010; Munoz-Price et al., 2013). The global increase in such carbapenemase-producing *Enterobacteriaceae* has resulted in increased use of colistin with the risk of emerging resistance. The development of colistin resistance is also directly linked with the agricultural use of human antibiotics, where some countries have actively used colistin in animal production (Hao et al., 2014).

Colistin belongs to the family of polymyxins, cationic polypeptides, with broad-spectrum activity against Gram-negative bacteria, including most species of the family of *Enterobacteriaceae*. The two polymyxins currently in clinical use are polymyxin B and polymyxin E (colistin), which differ only by one amino acid from each other and have comparable biological activity. The mechanism of resistance to polymyxins is modification of lipid A, resulting in a reduction of polymyxin affinity (Liu et al., 2016). The polymyxin resistance can be traced back to the plasmid mediated *mcr-1* gene. The plasmid carrying the *mcr-1* is already characterized and can be mobilized by conjugation (Liu et al., 2016). The prevalence of *mcr-1* in *E. coli* from livestock and food in Germany is documented by Irrgang et al. (2016), with the highest prevalence of *mcr-1* positive isolates of *Enterobacteriaceae* from poultry food chains. Another report documented the presence of *mcr-1* carrying plasmids in pig slurry in Estonia (Brauer et al., 2016). Furthermore, Ovejero et al. (2017) was able to isolate 30 *mcr-1* positive isolates from sewage of two WWTP in Spain. Their analysis suggested, that *mcr-1* in Spain besides *E. coli* has successfully transferred to *K. pneumoniae*. Bernasconi et al. (2016) also detected the *mcr-1* gene in stool samples of travelers returning from India. This indicates the fact that humans can get colonized by *mcr-1* resistant bacteria which are acquired through the food chain or from other environmental sources.

We report the prevalence of the plasmid mediated colistin resistance gene *mcr-1* together with other clinically relevant antibiotic resistance genes during wastewater treatment at municipal WWTPs in Germany. Quantitative PCR (qPCR) was used for the detection of the antibiotic resistance genes in native wastewater populations aiming on a microbiological risk characterization concerning the dissemination of colistin and other resistance genes into the adjacent aquatic environment.

## Materials and methods

### Sampling sites

A number of seven different German WWTPs were investigated. These WWTPs possess different population equivalents with WWTP-1 440,000, WWTP-2 210,000, WWTP-3 23,500, WWTP-4 46,000, and WWTP-5 26,000, WWTP-6 45,000, and WWTP-7 8,000 (Table 1). All WWTPs performed mechanical separation, followed by activated sludge treatment, and sedimentation steps.

**Table 1:** List of all investigated wastewater treatment plants with their respective specifications (unk.: unknown).

		**Capacity [p.e.]**	**Quantity [m^3^/d]**	**Temp [°C]**	**pH**	**Conductivity [μs/cm]**	**COD [mg/L]**	**NH_4_-N [mg/L]**	**NO_3_-N [mg/L]**	**NO_2_-N [mg/L]**	**TN_b_ [mg/L]**	**TotalP [mg/L]**
WWTP1	Influent	440,000	112,501	15.5	7.9	1,241	441	35.5	unk.	unk.	36.5	6.04
	Effluent		unk.	15.6	7.7	833	12.0	0.1	unk.	unk.	4.0	0.13
WWTP2	Influent	23,500	699	6.5	7.33	1,413	1,033	64.6	0.61	0.112	99.0	14.2
	Effluent		unk.	6.5	6.7	1,044	44.0	1.5	2.18	0.064	unk.	0.55
WWTP3	Influent	46,000	3,496	10.0	8.0	2,480	987	75.6	1.15	1.16	110.0	5.69
	Effluent		3,207	7.0	7.9	2,250	41.6	0.4	1.48	0.028	4.2	0.62
WWTP4	Influent	26,000	1,341	6.1	8.4	1,425	1,561	20	16.3	9.4	84.0	14.1
	Effluent		1,341	5.8	7.2	1,221	48.6	0.05	0.18	0.03	2.4	0.41
WWTP5	Influent	45,000	1,430	19.5	7.6	1,718	1,584	52.6	0.7	0.580	110.0	12.4
	Effluent		1,683	17.3	7.6	1,355	22.0	0.02	6.48	0.030	7.9	0.08
WWTP6	Influent	210,000	30,527	8.0	7.83	1,451	1,258	55.6	0.894	0.083	96.9	14.3
	Effluent		30,527	8.6	7.32	956	31.8	1.1	6.89	0.153	10.2	0.32
WWTP7	Influent	8,000	480	11.0	8.44	unk.	720.0	68.6	1.35	0.356	88.2	11.4
	Effluent		480	10.6	7.78	1,808	26.7	1.9	1.25	0.257	6.0	0.98

Whereas, WWTP-1 and WWTP-2 treat wastewater from urban areas including clinical, household, and industrial wastewaters, the wastewaters of the other 4 WWTPs are mainly affected by agricultural catchment areas including animal and food farming. WWTP 7 only treats municipal wastewater without affection of agriculture or industry.

Sampling points were arranged at the influents of WWTPs after mechanical separation and at the effluent sites after sedimentation tanks before the purified wastewater is released into the receiving bodies.

### Sample preparation and DNA extraction

Volumes of about 100 mL from the 24 h-composite of the WWTP influent samples and about 300 mL of the WWTP effluent samples from the 24 h-composite sample were used for DNA extraction. Wastewater samples were filtered using polycarbonate membranes with a diameter of 47 mm and a pore size of 0.2 μm (Whatman Nuclepore Track-Etched Membranes, Sigma-Aldrich, Munich, Germany). DNA extraction was performed using the Fast DNA spin kit for soil (MP Biomedical, Illkrich, France) utilizing the lysing matrix E according to the manufacturer's protocol for wastewater. The quantities and purities of the DNA extracts were measured by means of the Qubit fluorimetric quantitation (Thermo Scientific, Waldham, USA).

The yield of DNA obtained from the influent water samples ranged from 71 to 244 μg per mL (*n* = 14) and from 15 to 143 μg per mL (*n* = 14) for the effluent water samples. To determine the abundances of 7 clinically relevant antibiotic resistant genes including the *mcr-*1 colistin resistance gene, and the 16S rRNA gene as well as the gene markers for some *Enterobacteriaceae* qPCR analyses were performed.

### Primer design, qPCR protocol, and evaluation

All primers and references are listed in Table 2, which do also contains the accuracy (*R*^2^) as well as efficiency (%) values for each qPCR detection system. More specifically, the *Escherichia coli* NRZ-14408 reference strain is carrying the *mcr-1* colistin resistance gene and was kindly provided from National Reference Center in Bochum, Germany. For *mcr-1* primer design the NCBI Primer BLAST software was used. For the quantification of the *mcr-1* gene in environmental water samples a colistin-resistant *E. coli* NRZ-14408 (hospital isolate, *mcr-1* positive) reference bacterium was used to create a calibration curve, which is shown in Supplementary Material. An *R*^2^ value of 0.999 and an efficiency of 97.6% were determined, indicating the high specificity of the *mcr-1* detection system.

**Table 2:** List of all used primer systems with their corresponding target gene, expected amplicon size, accuracy, and efficiency of the used calibration curves.

**Organism**	**Target gene**	**Primer**	**Sequence**	**Amplicon (bp)**	**Accuracy (*R*^2^)**	**Efficiency [%]**	**References**
**ANTIBIOTIC RESISTANCE GENES**							
*E. coli*	*mcr-1*	mcr1FP	GGGCCTGCGTATTTTAAGCG	183	0.999	97.6	this study
		mcr1RP	CATAGGCATTGCTGTGCGTC				
*E. coli*	*CTX-M*	S_CTX-MuF	CGCTTTGCGATGTGCAG	551	1.0	92.9	Paterson et al., 2003
		S_CTX-MuR	ACCGCGATATCGTTGGT				
*E. coli* pNorm	*CTX-M-32*	ctxm32-F	CGTCACGCTGTTGTTAGGAA	155	1.0	92.5	Stalder et al., 2014
		ctxm32-R	CGCTCATCAGCACGATAAAG				
*K. pneumoniae*	*CMY-2*	CMY-2 RT-F	CGTTAATCGCACCATCACC	172	0.998	89.9	Kurpiel and Hanson, 2011
		CMY-2 RT-R	CGTCTTACTAACCGATCCTAGC				
*S. hyointestinales*	*ermB*	ermB-F	TGAATCGAGACTTGAGTGTGCAA	71	1.0	99.7	Alexander et al., 2015
		ermB-R	GGATTCTACAAGCGTACCTT				
*E. coli* pNorm	*blaTEM*	qblaTEM-F	TTCCTGTTTTTGCTCACCCAG	112	0.999	100.8	Stalder et al., 2014
		qblaTEM-R	CTCAAGGATCTTACCGCTGTTG				
*E. coli*	*tetM*	S_tetM-F	GGTTTCTCTTGGATACTTAAATCAATCR	88	0.998	95.9	Peak et al., 2007
		S_tetM-R	CCAACCATAYAATCCTTGTTCRC				
**TAXONOMY GENES**							
*E. coli*	*yccT*	yccTFP	GCATCGTGACCACCTTGA	59	0.994	98.4	Clifford et al., 2012
		yccTRP	CAGCGTGGTGGCAAAA				
*K. pneumoniae*	*gltA*	gltAFP	ACGGCCGAATATGACGAATTC	68	0.998	97.4	Clifford et al., 2012
		gltARP	AGAGTGATCTGCTCATGAA				
*E. coli* pNorm	*16S rRNA*	331FP	TCCTACGGGAGGCAGCAGT	195	1.0	96.6	Stalder et al., 2014
		518RP	ATTACCGCGGCTGCTGG				
*A. baumannii*	*secE*	secEFP	GTTGTGGCTTTAGGTTTATTATACG	94	1.0	97.6	Clifford et al., 2012
		secERP	AAGTTACTCGACGCAATTCG				

The antibiotic resistance genes *blaTEM, CTX-M, CTX-M-32*, and *CMY-2* are directed against ß-lactam antibiotics, whereas the *tetM* resistance gene is coding for the resistance against tetracycline and the *ermB* gene mediates the resistance against erythromycin. All primers, reference strains, and quality values of each detection system are listed in Table 2. In addition calibration and melting curves are given in Supplementary Material. The abundances of these ARGs were quantified in all WWTP effluent wastewater samples via qPCR approach using different reference strains carrying the mentioned resistance genes.

The *yccT* gene present in *E. coli* DSM 1103 was used as taxonomic gene marker as well as the *gltA* gene from *K. pneumoniae* DSM 30104 (Clifford et al., 2012). Total DNA of pure culture was extracted with the DNA extraction kit for soil (MP Biomedical, Illkrich, France) and subsequently used for the generation of target specific calibration curves. 16S rRNA primers were used to quantify eubacterial rDNA in the water samples for normalization. Here, an already cloned fragment of the eubacterial 16S rRNA gene in the pNORM plasmid of *E. coli* DH5α was used (Stalder et al., 2014). Plasmid-DNA was extracted with the GeneJet Plasmid Miniprep Kit (Thermo Scientific, Waldham, USA) and was used for the generation of the calibration curve which in turn was used for the calculation of the 16S rRNA gene copy number in water samples.

The *mcr-1, ermB, tetM* resistance genes, and the four ß-lactamase genes (*CTX-M. CTX-M-32, CMY-2, blaTEM*), the ribosomal 16S rRNA gene for *Eubacteria*, and the specific taxonomic gene markers of *E. coli, K. pneumonia, and Acinetobacter baumannii* were quantified in a SYBR Green qPCR approach. Reactions were run in volumes of 20 μL, containing 10 μL Maxima SYBR Green/ROX qPCR Master Mix (2x) (Thermo Scientific), 8.2 μL of nuclease-free water (Ambion, Life technologies, Karlsbad, Germany), 0.4 μL of the respective primers (stock concentration 10 μM, Table 2), and 1 μL of template DNA (20 ng μL^−1^). The qPCR protocol comprised 10 min at 95°C for activation of the DNA polymerase followed by 40 cycles of 15 s at 95°C and 1 min at appropriate temperature for primer annealing and elongation (see Supplementary Material). Each water sample was analyzed in technical triplicates. To determine the specificity of the amplification, a melting curve was recorded by raising the temperature from 60 to 95°C (1°C every 10 s). Data analysis was performed by using the Bio-Rad CFX Manager software.

### Cell equivalent calculation

To calculate the gene copy number of the respective antibiotic resistance gene, the 16S rRNA gene, and taxon-specific gene marker, reference strains carrying the genetic targets of interest were used. With regard to the already known genome sizes of the reference bacteria it is possible to calculate the cell copies. The following equation with an average molecular weight for one base pair of about 650 g/mol, Avogardo's number with 6.022 × 10^23^ molecules/mol, and a converting factor of 10^9^ ng/g was used.

(1) $$\begin{array}{r} number\;of\;copies=\;\frac{\left(amount\;of\;DNA\;\left[ng\right]\right)*6,022*10^{23}}{\left(average\;size\;of\;genome\left[bp\right]\right)*\;10^{9}*650} \end{array}$$

Serial dilution of DNA stocks were prepared and used for calculation of the calibration curves starting with 500 ng DNA (see Supplementary Material). The coefficient of determination of all standard curves was above 0.994 in all experiments (Table 2), indicating minimal variability within the linear data range.

Using these curves, the measured Ct values of the *mcr-1* gene, the 16S ribosomal RNA gene or the taxon-specific gene markers from water samples can be used for copy number calculations (Alexander et al., 2015, 2016). The abundances of antibiotic resistance genes (ARGs) and opportunistic bacteria were quantified in each water sample and normalized to 100 ng total extracted DNA (cell equivalents per 100 ng DNA). In addition, the qPCR data was also normalized to the 16S rRNA gene abundances of the corresponding water samples.

### Data statistics and presentation

In total 14 biological samples from the influents and effluents of 7 WWTPs were obtained from two individual sampling campaigns in autumn and spring time. For data presentation the standardized box plot diagram was used displaying the distribution of data based on the five number summaries: minimum, first quartile, median, third quartile, and maximum. In the box plot the central rectangle spans the first quantile (*P* = 25) to the third quantile (*P* = 75). A segment inside the rectangle shows the median and whiskers above and below the box show the minimum and maximum values. Thus, the box plot displays the full range of variation (from min to max), the likely range of variation, and a typical value (the median). In addition, student's *t-*test calculations were performed to identify the significance of reduction of gene targets during conventional wastewater treatments.

### Propidium monoazide (PMA) treatment

PMA can enter dead or injured bacteria due to the loss of their membrane integrities, intercalate with the intracellular and extracellular DNA. The presence of the PMA-DNA complex blocks the polymerase activity at the target sites. In consequence, no PCR product is generated. The protocol is in accordance with that of our previous study (Villarreal et al., 2013) and based on the publications of Nocker et al. (2007a,b) and Nocker and Camper (2009), where the method was optimized also for natural mixed populations. To separate the living bacterial population from dead or injured bacteria during conventional wastewater treatment, the PhAST Blue Photo-Activation System (GenIUL, Barcelona, Spain) was used. After filtration of the influent and effluent wastewater samples originating from WWTP 1 (*n* = 3), polycarbonate membranes (Nucleopore) were submerged in 300 μL of a 25 μM propidium monoazide (PMA; Biotium, Hayward, California, USA) solution and put into a 1.5 mL colorless tube (SafeSeal tubes, Carl Roth, Karlsruhe, Germany). After incubation in the dark at 4°C for 5 min, samples were exposed to the LED light of the photoactivation system at 100% intensity for 15 min. After PMA treatment, samples were prepared for DNA extraction using the Fast DNA spin kit for soil according to the manufacturer's protocol for wastewater.

### Agarose gel electrophoresis and sequencing of *mcr-1* amplicons

The *mcr-1* amplicons were separated on a 1% agarose gel to examine the primers and PCR protocols for proper specificity. The results demonstrated that *mcr-1* positive water samples generated amplicons with the expected length of 183 bp (**Figure 2**). Sequence analyses confirmed the *mcr-1* identity (see Supplementary Material). For that, the *mcr-1* PCR products were purified with ExoSAPit (GE Healthcare, Munich, Germany) before sequencing. The sequencing reactions were performed in a 10 μL reaction volume, including 2 μL of Premix (BigDye® Terminator v1.1 Cycle Sequencing Kit, Applied Biosystems), 0.5 μL primer 517R (10 pM), and 1 μL of purified, 1:3 diluted PCR product. This volume was filled up to 10 μL with sterile water. The temperature profile of the sequencing reaction was as followed: 5 min at 96°C, 25 cycles of 10 s at 96°C, 5 s at 55°C, and 1 min at 60°C, afterwards cooling down to 4°C. For deleting excessive dye, the sequence products were purified with DyeEx2.0 Spin Kit (Qiagen, Hilden, Germany). Three microliters of this product were added to 12 μL of Hi-Di-Formamid (Applied Biosystems) and analyzed in the ABI Prism 310 Genetic Analyser (Applied Biosystems) using POP4 Polymer (Applied Biosystems) and 47 cm × 50 μm capillaries (Applied Biosystems) according to the manufacturer‘s instructions. For database analyses, NCBI (National Center for Biotechnology Information) Blast alignments were performed.

## Results

### Feasibility of the detection system targeting *mcr-1* resistance gene in wastewater population demonstrated at WWTP-1

**Figure 1:**
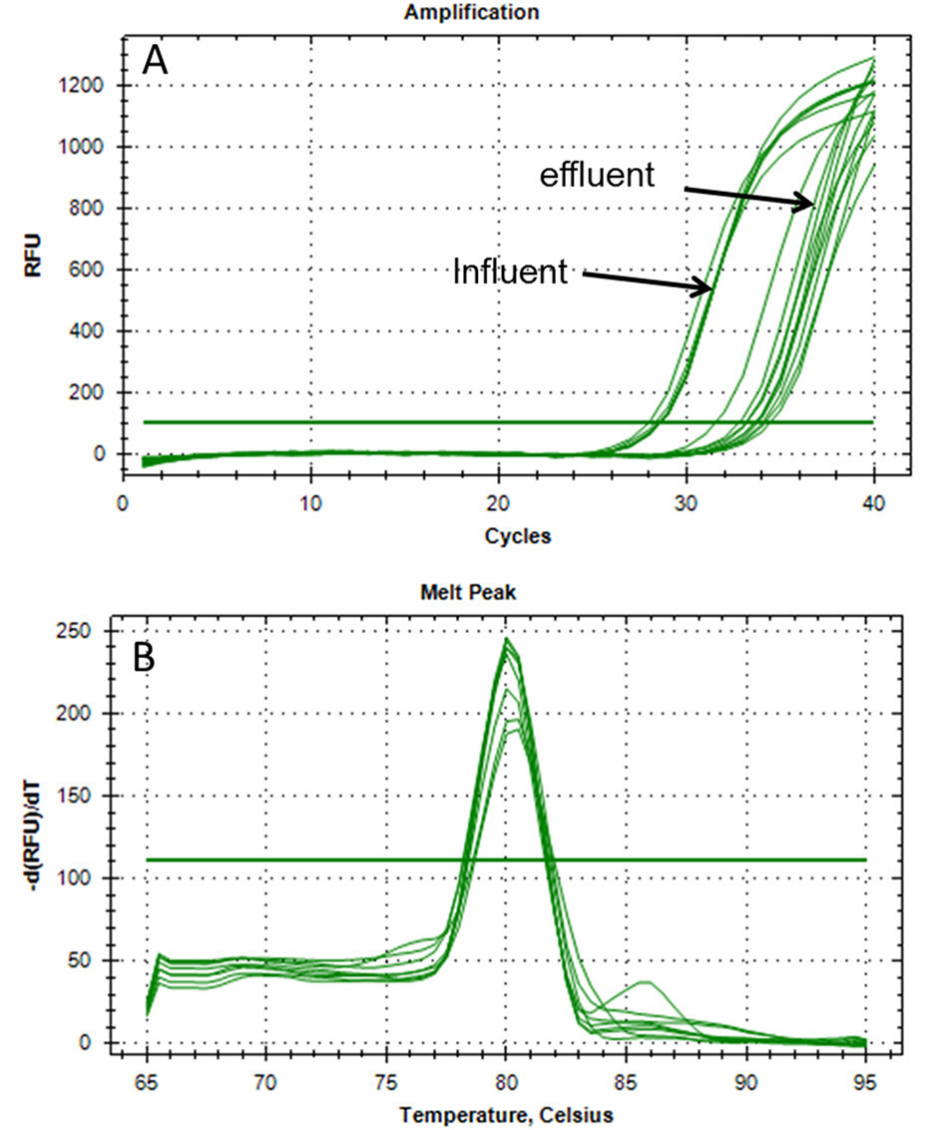
qPCR fluorescence signal of *mcr-1* amplification from total extracted DNA. All samples were from influent and effluent wastewater of the WWTP-1. No abnormal amplification results could be observed **(A)**. Change of fluorescence units over increasing temperature (melting curve) of *mcr-1* amplicons. No unexpected shoulders were detected above the threshold **(B)**.

**Figure 2:**
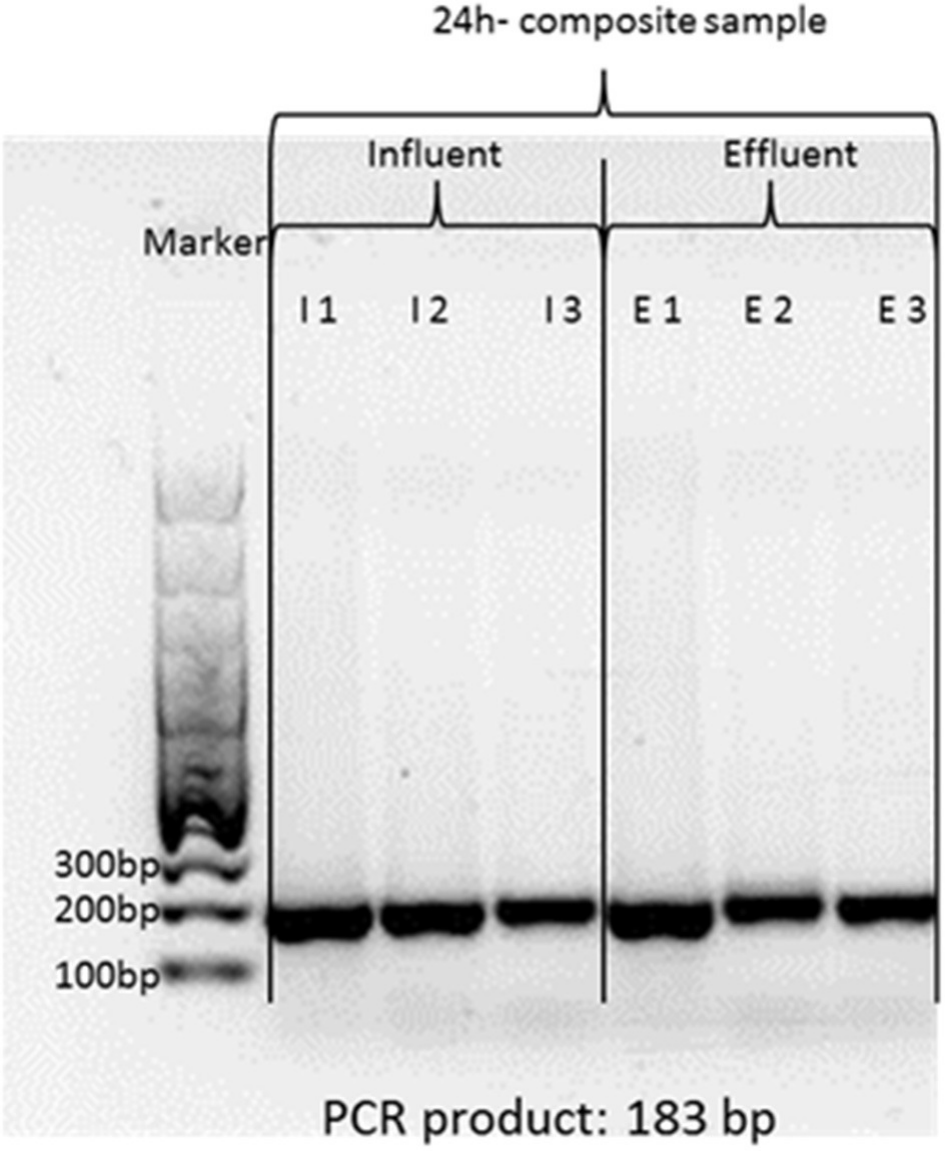
One percent agarose gel of *mcr-1* amplicons from influent and effluent of WWTP-1. Theoretical amplification length is 183 base pairs. The gel showed only one specific band at the expected length.

The accuracy of the novel qPCR detection system targeting the *mcr-1* colistin resistance gene in wastewater samples was demonstrated with the extracted total DNA of original water samples. Here, total DNA from the WWTP-1 with the highest population equivalent of 440.000 was used. Neither irregular amplification results were observed during qPCR nor unexpected shoulders seen during melting curve analyses (Figures 1A,B). After qPCR amplification the amplicons were separated in an agarose gel and resulted in one specific DNA band positioned at the expected product size of 183 bp (Figure 2). The amplicons were also sequenced. The evaluation of the BLAST search confirmed the 99% identity with the *mcr-1* resistance gene from *E. coli* (LC191581), *K. pneumoniae* (KX377410), *Salmonella enterica* (KX257482), and *Cronobacter sakazakii* (KX505142) strain. As a first result of this study it could be shown that this newly developed qPCR system is suited for the reliable quantification of the *mcr-1* gene in wastewater populations.

The Ct-values from qPCR were used for cell equivalent calculations normalized to 100 ng total DNA, which is a relative quantification related to the population microbiome. It is independent from the sample volume. Additionally, the cell equivalents from the calibration curve were normalized to the 16S rRNA gene copies as an alternative eubacterial biomass gene marker. It became obvious that the *mcr-1* gene was present in influent samples of WWTP-1 with a calculated abundance of 8.11 × 10^1^ cell equivalents per 100 ng total DNA. The median values of cell equivalent per 100 ng DNA of the effluent water samples were quantified with 7.0 × 10^1^. Alternatively, the cell equivalents were also referred to 16S rRNA gene copies and corresponded with 2.64 × 10^−8^ cell equivalents in the influent and 1.30 × 10^−9^ cell equivalents in the effluent water samples, respectively. The data from WWTP-1 is indicating the stable presence of *mcr-1* gene within the microbiome of the wastewaters of this large WWTP.

Additionally, influent and effluent wastewater samples of WWTP-1 were treated with PMA to discriminate living bacteria from dead bacteria or extracellular DNA (eDNA) prior to DNA extraction. The presence of eDNA, which might be released from dead or injured bacteria, is known to be a promoting factor for horizontal gene transfer (HGT) (Davies et al., 2006; Aminov, 2011). In case of the colistin resistance the *mcr-1* gene is located on a conjugative plasmid, which is already described to transfer the colistin resistance among *Enterobacteriaceae* (e.g., Liu et al., 2016). After normalization to 100 ng DNA and 16S rRNA gene copies slightly reduced cell equivalents were found in case of PMA treatment in influent and effluent samples (influent PMA treated: 6.2 × 10^0^ cell equivalents per 100 ng DNA and 8.92 × 10^−8^ per 16S rRNA gene copy; effluent PMA treated: 6.2 × 10^0^ cell equivalents per 100 ng DNA; and 3.66 × 10^−8^ cell equivalents per 16S rRNA gene copy). Comparing PMA-treated with untreated samples of the influent and effluent in maximum one order of magnitude difference became obvious. These results are indicating the presence of low amounts of eDNA or distinct numbers of injured/dead bacteria in wastewater samples, which might have a relevance for transformation and therefore HGT.

Nevertheless, it could be shown that the *mcr-1* colistin resistance gene is present in water samples from WWTP-1 and still living bacteria carrying the *mcr-1* gene are released to the receiving adjacent body.

### Comparing different WWTPs according to the presence of *mcr-1* gene

**Figure 3:**
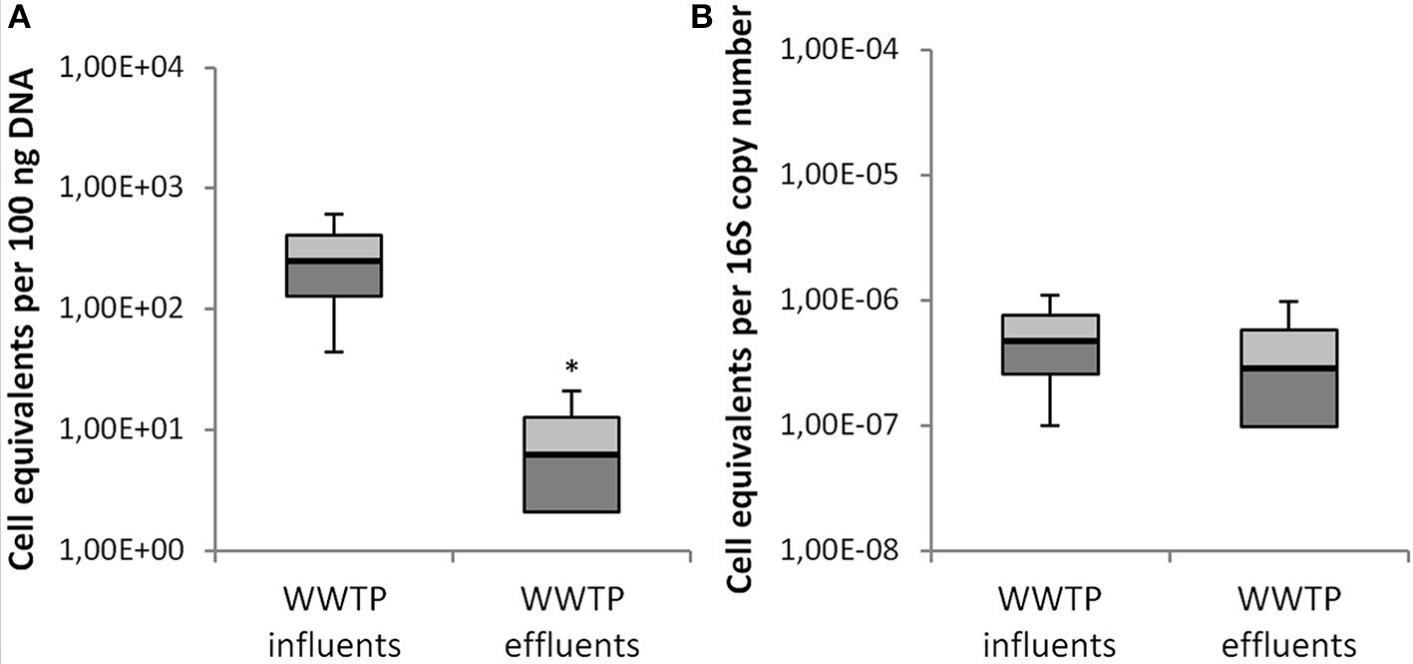
Abundance of *mcr-1* cell equivalents in influent and effluent of all 7 WWTP per 100 ng DNA **(A)** and per 16S rRNA copy number **(B)**. Displayed are the median values as well as the quantils [*p* = 0.25 (dark), *p* = 0.75 (bright)] and the standard deviations (*n* = 14). Significance of reduction is assessed by student's *t-*test with *p* ≤ 0.05 and are indicated by asterisk.

As mentioned before, the influent and effluent wastewaters of 6 additional WWTPs were investigated for the abundance of the *mcr-1* gene coding for the colistin resistance in *Enterobacteriaceae*. In contrast to WWTP-1 the population equivalents of these WWTPs were much lower ranging from 8,000 to 210,000 p.e. The *mcr-1* gene was quantified in the influent wastewater samples of all six WWTPs. These *mcr-1* positive tested WWTPs treat urban and rural wastewaters including hospitals, livestock, and food industries. Here, the cell equivalents ranged from 4.45 × 10^1^ to 2.01 × 10^2^ in 100 ng total DNA, and 9.89 × 10^−8^ to 3.44 × 10^−7^ per 16S rRNA gene copy number (Figure 3).

Compared to WWTP-1 (440,000 p.e.), the influent waters of these WWTPs showed higher abundances of the *mcr-1* gene (Figure 3), which might result from the agricultural or food industry impacts. Even in influent samples of the smallest WWTP-7 (8,000 p.e.) *mcr-1* gene copies were detected despite the fact that no hospitals and no intensive animal farms or food industry released wastewaters to this WWTP.

Besides WWTP-1, the *mcr-1* gene was detected in the effluent water samples of WWTP-2 and WWTP-3 with 8.4 × 10^0^ to 1.3 × 10^1^ cell equivalents per 100 ng total DNA and 3.88 × 10^−7^ and 1.55 × 10^−7^ per 16S rRNA.

Hence, the *mcr-1* gene was detected both in the influent waters of all seven WWTPs and in the effluent water samples of WWTP-1, WWTP-2, and WWTP-3.

These WWTPs differed in population equivalents (Table 1), which gives hints that the dimension of the WWTPs does not significantly impact the survival and persistence of *mcr-1* gene carrying bacteria. For the *mcr-1* gene a maximum reduction of 1 Log scale was determined in WWTP-1, WWTP-2, and WWTP-3.

### Detection of other clinically relevant antibiotic resistance genes

**Figure 4:**
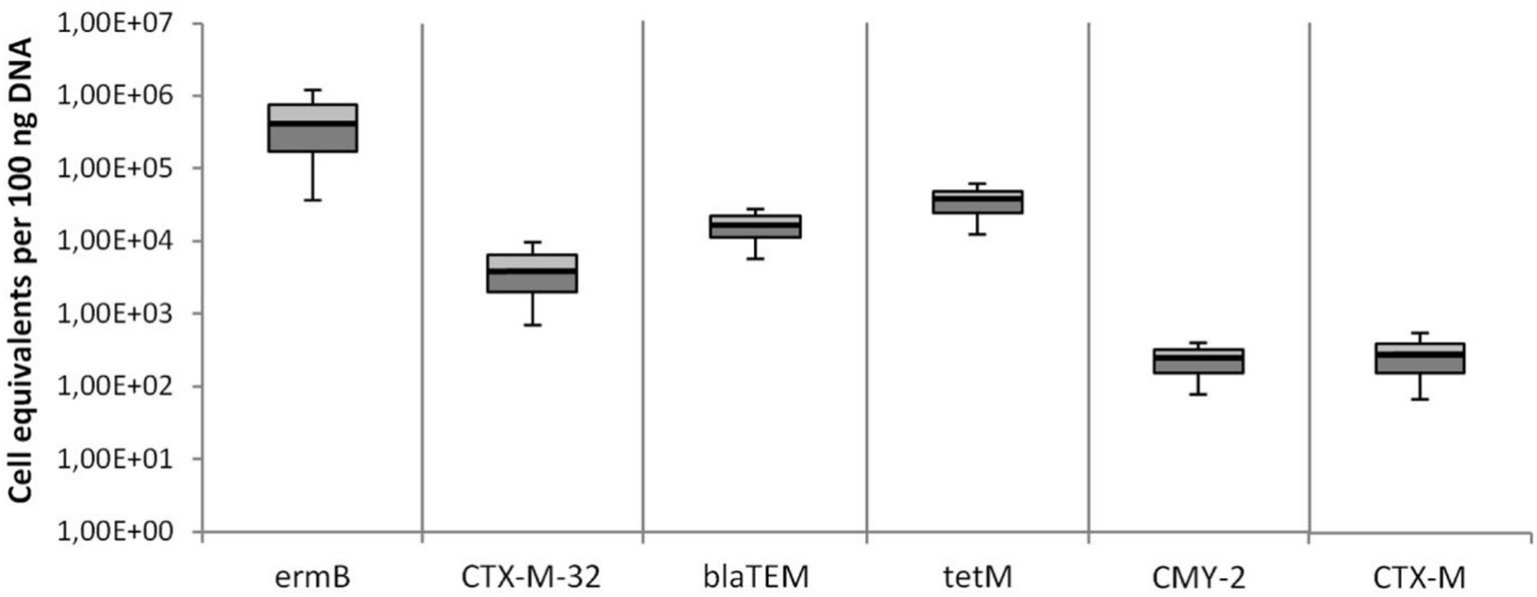
Abundances of antibiotic resistance cell equivalents per 100 ng DNA derived from total extracted DNA of all 7 WWTPs effluents. Erythromycin resistance was detected by *ermB* gene abundance, ß-lactam resistance genes were detected by *CTX-M32, blaTEM, CMY-2*, and *CTX-M* gene abundances, and tetracycline resistance was detected by *tetM* gene abundance. Displayed are the median values as well as the quantils [*p* = 0.25 (dark), *p* = 0.75 (bright)] and the standard deviations (*n* = 14).

**Figure 5:**
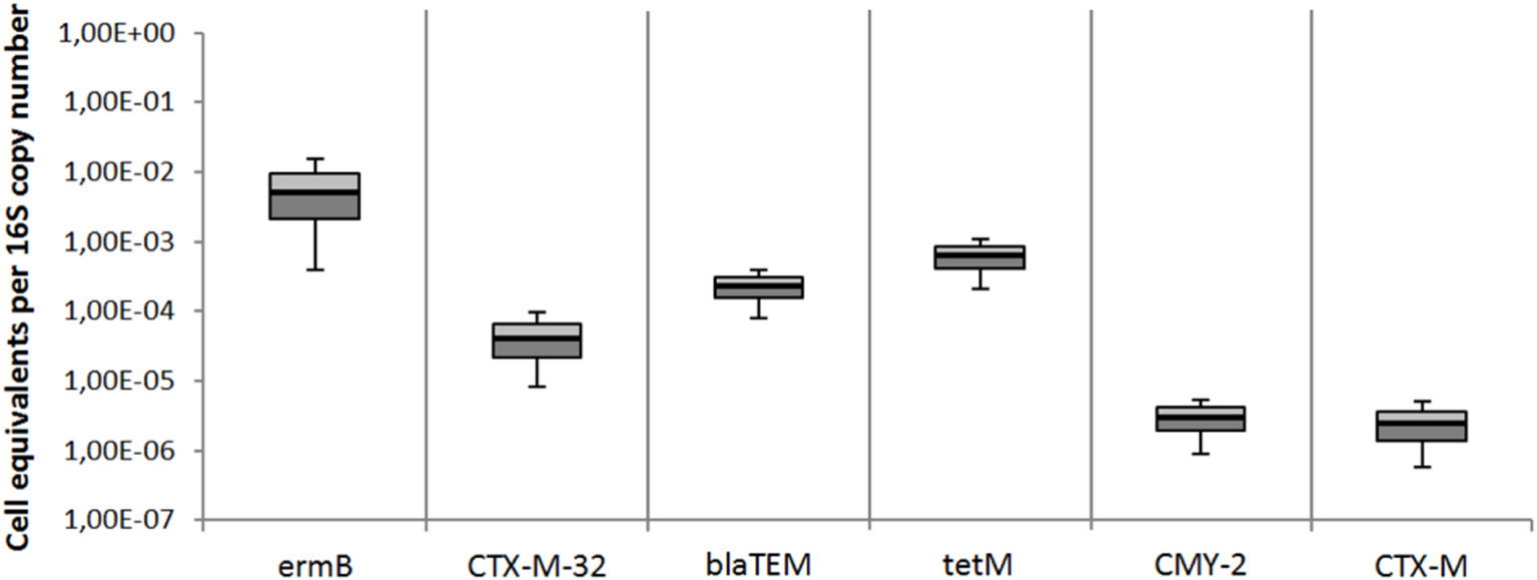
Abundances of antibiotic resistance genes per 16S rRNA gene copy number derived from total extracted DNA of all 7 WWTPs effluents. Erythromycin resistance was detected by *ermB* gene abundance, ß-lactam resistance genes were detected by *CTX-M32, blaTEM, CMY-2*, and *CTX-M* gene abundances, and tetracycline resistance was detected by *tetM* gene abundance. Displayed are the median values as well as the quantils [*p* = 0.25 (dark), *p* = 0.75 (bright)] and the standard deviations (*n* = 14). Student's *t-*test values were all >0.05.

Besides the *mcr-1* colistin resistance gene, 6 other clinically relevant antibiotic resistance genes were quantified in effluent wastewater samples of the 7 WWTPs. Both the relative quantification and the cell equivalents per 16S rRNA gene copy number are shown in Figures 4, 5. The targeted genes are the *ermB* gene coding for the erythromycin resistance, the tetracycline resistance gene *tetM*, and 4 different ß-lactam resistance genes (*CTX-M*-32, *blaTEM, CMY*-2, and *CTX-M*). All resistance genes were distinctly present in all wastewater effluents samples. The most abundant resistance gene was *ermB* with a median value of 2.39 × 10^5^ cell equivalents in 100 ng DNA or 3.08 × 10^−3^ cell equivalents per 16S rRNA gene copy number. The second most abundant gene was the tetracycline resistance gene *tetM* with 1.26 × 10^4^ cell equivalents in 100 ng DNA and 1.68 × 10^−4^ per 16S rRNA gene copy number. The abundances of the *blaTEM*, and *CTX-M*-32 genes were found in a similar range slightly below the *tetM* gene. The median values for the *CMY-2* and *CTX-M* ß-lactamase genes were detected in a lower abundance of 1.0 × 10^2^ in 100 ng DNA and 8.7 × 10^−7^ per 16S rRNA gene copy number.

In comparison to *mcr*-1 gene abundances, these resistance genes were much more frequently found and were quantified in higher concentrations in all wastewater effluents released to the aquatic environment. Data from the influent samples of WTTPs demonstrated a reduction of the mentioned resistance genes ranging from 1 Log to maximum of 2 Logs during wastewater treatment (data not shown), which is in accordance with the data targeting the *mcr-1* gene.

### Opportunistic pathogens in the wastewater samples

The abundance of specific taxonomic gene markers was quantified targeting *A. baumannii* and the *Enterobacteriaceae K. pneumoniae* and *E coli*. These three bacterial species are described to belong to the network of horizontal gene transfer with clinical relevance. All of them are opportunistic pathogens and were shown to hold resistances to colistin by previous studies (Hua et al., 2017; Jeannot et al., 2017). To address the growing concern of emerging colistin resistant pathogens, the abundance of these possible carrier bacteria are also analyzed in the German WWTP of this study.

**Figure 6:**
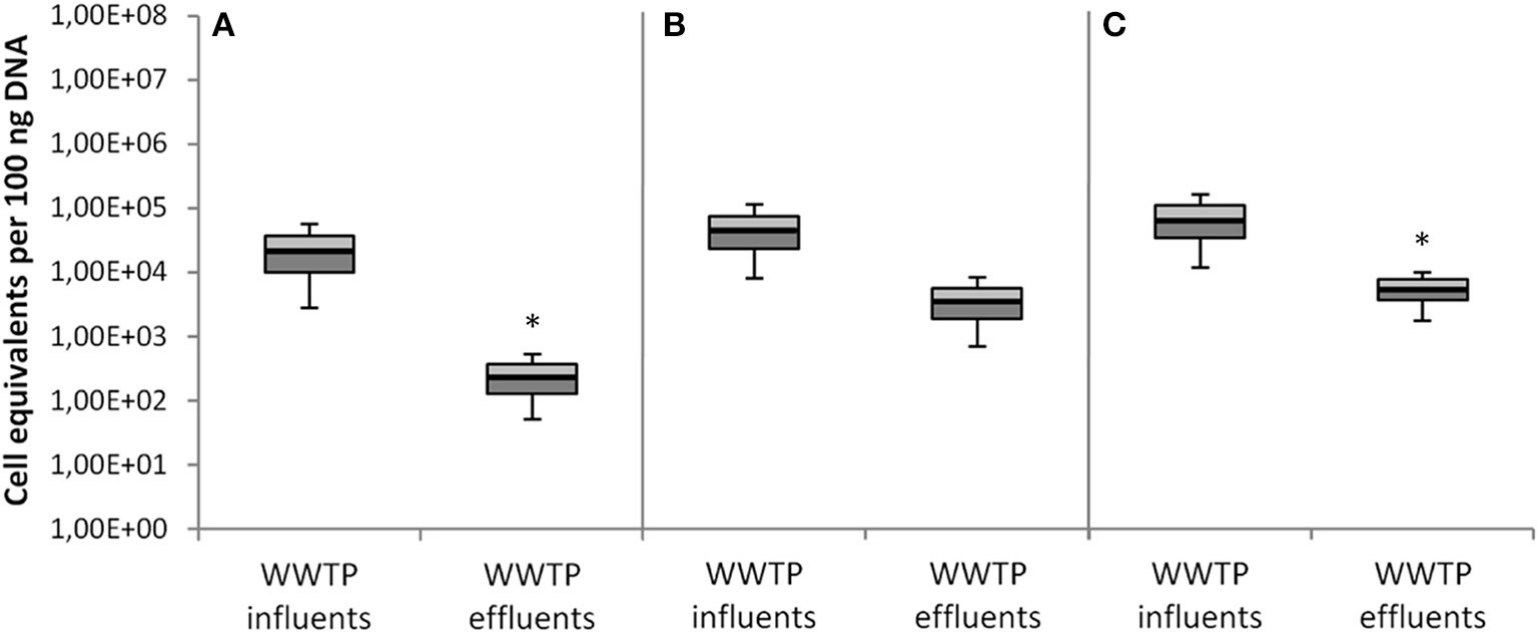
Abundances of taxonomical marker genes per 100 ng of total extracted DNA from all 7 WWTPs. **(A)** *A. baumannii* was detected by *secE* gene abundance, **(B)** *K. pneumoniae* was detected by *gltA* gene abundance, and **(C)** *E. coli* was detected by *yccT* gene abundance. Displayed are the median values as well as the quantils [*p* = 0.25 (dark), *p* = 0.75 (bright)] and the standard deviations (*n* = 14). Significance of reduction is assessed by student's *t-*test with *p* ≤ 0.05 and are indicated by asterisk.

**Figure 7:**
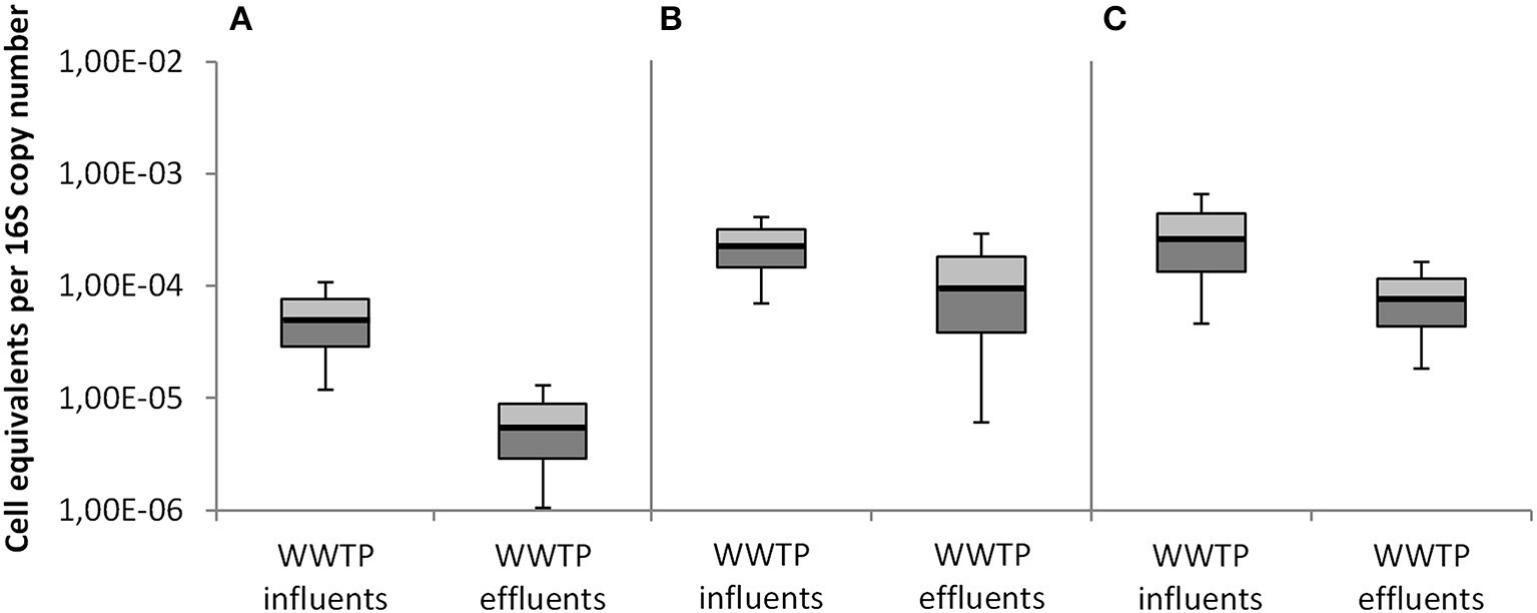
Abundances of taxonomical marker genes per 16S rRNA gene copy number derived from total extracted DNA of all seven WWTPs. **(A)** *A. baumannii* was detected by *secE* gene abundance, **(B)** *K. pneumoniae* was detected by *gltA* gene abundance, and **(C)** *E. coli* was detected by *yccT* gene abundance. Displayed are the median values as well as the quantils [*p* = 0.25 (dark), *p* = 0.75 (bright)] and the standard deviations (*n* = 14). Student's *t*-test values were all >0.05.

The Figures 6, 7 summarize the data of all seven investigated WWTPs (influent/effluent) according to the two different normalization approaches, i.e., per 100 ng total DNA (Figures 6A–C) and per 16S rRNA gene copy (Figures 7A–C). Both figures show the abundances of taxonomic gene markers specific for *A. baumannii* (*sec*E), *K. pneumoniae* (*glt*A), and *E. coli* (*ycc*T). Calibration curves and melting curve analyses for each parameter are documented in Supplementary Material. The obtained Ct values matched with the linear ranges of the calibration curves.

The median values of the influent samples were calculated for the specific taxonomic genes with 1.12 × 10^4^ for *A. baumannii*, 2.15 × 10^4^ for *K. pneumoniae*, and 2.96 × 10^4^ for *E. coli* cell equivalents per 100 ng total DNA. Decreased median values per 100 ng DNA resulted from the effluent samples for all three target genes, i.e., 1.05 × 10^2^ for *A. baumannii*, 1.60 × 10^3^ for *K. pneumoniae*, and 1.80 × 10^3^ for *E. coli* (Figure 6). In total, the abundances of these taxonomic gene markers decreased during conventional wastewater treatment with 1–2 orders of magnitudes, but are still present in significant amounts in effluent samples.

Figure 7 illustrates the abundances of taxonomical marker genes referred to the 16S rRNA copy number. The median values were analyzed and resulted in 2.17 × 10^−5^ cell equivalents for *A. baumannii*, 8.25 × 10^−5^ for *K. pneumoniae*, and 1.3 × 10^−4^ for *E. coli* in the influents samples. The investigation of cell equivalents in the effluent samples resulted in a median of 2.60 × 10^−6^ for *A. baumannii*, 5.86 × 10^−5^ for *K. pneumoniae*, and 3.29 × 10^−5^ for *E. coli*.

## Discussion

To the best of our knowledge, this is the first study that demonstrated the occurrence of the colistin resistance *mcr-1* gene in bacterial populations of wastewater. The *mcr-1* gene was detected in influent samples of all seven WWTPs and was not eliminated during wastewater treatment reaching the aquatic environment. The overall abundances, expressed as cell equivalents per 100 ng DNA or eubacterial 16S rRNA gene copy numbers are still low and live/dead analyses demonstrated that the *mcr-1* gene was present in living bacteria, released to receiving bodies.

The colistin resistance can be traced back to the plasmid carrying the *mcr-1* gene (Liu et al., 2016). This plasmid is already characterized and can be mobilized by conjugation. Regarding wastewater systems a potential high risk for horizontal gene transfer of the *mcr-1* carrying conjugative plasmid is present, since many factors promoting horizontal gene transfer are occurring in water samples from WWTPs (Bellanger et al., 2014). The combination of high cell densities in activated sludge tanks, increased nutrient availability, co-selections by heavy metals and selective pressures like complex mixtures of low-concentrated xenobiotics (e.g., antibiotics, biocids, disinfectants, pharmaceuticals) is hypothesized to promote horizontal gene transfer and therefore the persistence of certain antibiotic resistant bacteria in wastewater environments (Rizzo et al., 2013; Berendonk et al., 2015). This is of major concern because it has been shown, that some human pathogens are involved in high rates of horizontal gene transfer like *K. pneumoniae* (Hu et al., 2016; Navon-Venezia et al., 2017) and could enhance the dissemination of the *mcr-1* plasmid. Potential *mcr-1* carrying opportunistic pathogens, i.e., *A. baumannii, E. coli*, and *K. pneumoniae* were quantified in higher abundances even at effluent sampling sites.

Cultivation experiments of specific *mcr-1* positive *Enterobacteriaceae* from wastewater populations failed due to the low abundances of the target bacteria and due to the high abundance of accompanying bacterial flora, which overgrew the agar plates supplemented with polymyxin for colistin resistant *Enterobacteriaceae*.

The dissemination of colistin resistant bacteria via municipal wastewaters is only one way of dissemination of these antibiotic resistant bacteria. Other important dissemination pathways of antibiotic resistant pathogens were already mentioned (Vaz-Moreira et al., 2014). It needs to be studied how far the abundance of the *mcr-1* gene target is changing over times. An increasing number of this gene target, especially in clarified wastewater samples released to the aquatic environment, is then directly linked with an increasing microbiological risk potential and would underline the requirements of additional treatment steps to eliminate these opportunistic and antibiotic resistant bacteria.

According to the results of this study, the release of antibiotic resistance genes and opportunistic pathogens from WWTPs in significant amounts to the environment was demonstrated and underlines the requirement of an expanded wastewater treatment (Rizzo et al., 2013; Alexander et al., 2015). Most available data for the selected antibiotic resistance genes derived from clinical studies. Here, the occurrence of *blaTEM, CTX-M, CTX-M-32*, and *CMY-2*, all present in the investigated wastewater samples, leads to resistance against ß-lactam antibiotics. ß-lactams made up 30% of all prescript antibiotics in Germany (ECDC, 2015). As a consequence, the high prescription may lead to an increase resistance gene evolution and finally dissemination to the wastewaters. The *blaTEM* gene, quantified in high abundance of the WWTP effluents under investigation, is responsible for high percentages of ampicillin resistance in *E. coli* and is also prevalent in *K. pneumoniae* (Cooksey et al., 1990). It is considered to be a precursor of the extended-spectrum-ß-lactamases (ESBL) (Emery and Weymouth, 1997). *CTX-M* type ß-lactamases are the most widespread types of ESBL (Bonnet, 2004). Plasmids containing *blaCTX-M* genes often contain also *blaTEM* as well as *blaOXA* genes (Poirel et al., 2005). The *CTX-M-32* gene mediates a high resistance in pseudomonads and renders ceftazidime completely ineffective (Fernández et al., 2007). In addition, the *CMY-2* gene represents a resistance gene against carbapenems. Carbapenems are used when therapies with other ß-lactam antibiotics failed (Bauernfeind et al., 1996).

It became obvious, that these ß-lactam resistance genes were still present in all effluent samples indicating an insufficient reduction during conventional wastewater treatment. This was also demonstrated by the *t-*test calculations indicated by values with *p* < 0.05. In addition, the e*rmB* gene provides resistance against erythromycin, which is a macrolide antibiotic used as substitute for penicillin for patients with allergies against penicillin or infections with resistant bacteria to ß-lactams (Jelić and Antolović, 2016). Macrolides are used for respiratory or gastrointestinal infections and erythromycin made up to 16% of all antibiotics in Germany (ECDC, 2015). Finally, the *tetM* resistance gene mediates resistance against tetracycline, the second most common antibiotic in the world and currently used as food additive in animal feeding (Gu and Karthikeyan, 2005). Tetracycline and erythromycin often reach WWTP in sub-letal concentrations which might promote the spread of the corresponding resistances through horizontal gene transfer (Alexander et al., 2015).

In consequence, the integration of oxidative, chemical-physical, or membrane-based technologies for an adjusted wastewater treatment is a necessity to interrupt dissemination pathways of ARBs and ARGs to the aquatic environments. The fate of antibiotic resistant and opportunistic pathogens in different environmental habitats depends on species and genetic backgrounds. Some opportunistic pathogens are highly adaptable to stress situation and are able to activate effective stress responses to survive adverse growth conditions (nutrient limitation, low temperatures, etc.; Alexander et al., 2015, 2016). Hence, these microorganisms are able to persist in uncomfortable situation and will not die. The risk of contamination via direct contact of humans with insufficient cleaned wastewater or via vegetables irrigated with contaminated water (direct or indirect re-use) can't be excluded. The degree of environmental contamination via wastewater effluents depends on (1) the densities of WWTPs in a specific area, (2) the dimension of conditioned wastewaters released to the receiving bodies, and (3) the catchment area of the WWTP. It became clear by this study, that the size of the WWTP is not an adequate parameter to discuss a possible reduction of antibiotic resistance. The reduction was limited to only 1 to a maximum of 2 Logs during conventional treatment in all WTTPs ranging from low to high population equivalents. Thus, WWTPs should include an effective disinfection technique to avoid an environmental contamination with clinically relevant microbes to stop the dissemination and evolution of antibiotic resistances. Such an effort would also contribute to an effective protection of reservoirs for drinking water production even in industrialized countries. In fact, no national or international regulations or threshold values are available that mention the dissemination of clinically relevant microbes including antibiotic resistances via WWTPs to the aquatic environments. In fact, integrated evaluation concepts to assess the efficiency of advanced wastewater treatment processes for the elimination of antibiotic resistant bacteria and micro-pollutants are already published (Ternes et al., 2017) and are supposed to be a useful tool to initialize regulation processes.

## Author contributions

TS: Experimental organization, preparing the manuscript. NH, JA, and FS: Authors performed experiments and generate the scientific data. Co-authors of the manuscript. CH: Local support at the municipal wastewater treatment plants, sampling procedures, providing WWTP specific data. ER: Providing sequencing data.

### Conflict of interest statement

The authors declare that the research was conducted in the absence of any commercial or financial relationships that could be construed as a potential conflict of interest.
