# Supplementary material for: Occurrence of the mcr-1 Colistin Resistance Gene and other Clinically Relevant Antibiotic Resistance Genes in Microbial Populations at Different Municipal Wastewater Treatment Plants in Germany
Source: Front Microbiol. 2017 Jul 11;8:1282. doi: 10.3389/fmicb.2017.01282 (PMC5504345; doi:10.3389/fmicb.2017.01282)
Supplement: Supplementary file 1 [file DataSheet1.DOCX]

Dear Dr. David Graham. dear editorial board of Frontiers in Microbiology!

# Please find enclosed the Supporting Information of the revised manuscript: “Occurrence of the mcr-1 colistin resistance gene and other clinically relevant antibiotic resistance genes in microbial populations at different municipal wastewater treatment plants in Germany.” with the authors Hembach Norman, Schmid Ferdinand, Alexander Johannes, Hiller Christian, Rogall Eike Thorben, and Schwartz Thomas*.

*Corresponding author

Number of pages: 12

Number of figures: 20

Authors:

Norman Hembach

Karlsruhe Institute of Technology (KIT), Institute of Functional Interfaces (IFG), Bioengineering and Biosystems Department

Herrmann von Helmholtz Platz 1, 76344 Eggenstein-Leopoldshafen, Germany

E-Mail: norman.hembach@kit.edu; phone: +49 721 608-26802; fax: +49 721 608-26858.

Ferdinand Schmid

Karlsruhe Institute of Technology (KIT), Institute of Functional Interfaces (IFG), Bioengineering and Biosystems Department

Herrmann von Helmholtz Platz 1, 76344 Eggenstein-Leopoldshafen, Germany

E-Mail: ferdinand.schmid@kit.edu; phone: +49 721 608-26802; fax: +49 721 608-26858.

Johannes Alexander

Karlsruhe Institute of Technology (KIT), Institute of Functional Interfaces (IFG), Bioengineering and Biosystems Department

Herrmann von Helmholtz Platz 1, 76344 Eggenstein-Leopoldshafen, Germany

E-Mail: [johannes.alexander@kit.edu](mailto:johannes.alexander@kit.edu); phone: +49 721 608-26802; fax: +49 721 608-26858.

Christian Hiller

Zweckverband Klärwerk Steinhäule

Reinzstraße 1, 89233 Neu-Ulm, Germany

E-Mail: c.hiller@zvk-s.de; phone: +49 731 97 97 2 137; fax: +49 731 97 97 2 47.

Eike Thorben Rogal

Karlsruhe Institute of Technology (KIT), Institute of Functional Interfaces (IFG), Bioengineering and Biosystems Department

Herrmann von Helmholtz Platz 1, 76344 Eggenstein-Leopoldshafen, Germany

E-Mail: eike.rogal@kit.edu; phone: +49 721 608-26802; fax: +49 721 608-26858.

Prof. Dr. Thomas Schwartz*

Karlsruhe Institute of Technology (KIT), Institute of Functional Interfaces (IFG), Bioengineering and Biosystems Department

Herrmann von Helmholtz Platz 1, 76344 Eggenstein-Leopoldshafen, Germany

E-Mail: [thomas.schwartz@kit.edu](mailto:thomas.schwartz@kit.edu); phone: +49 721 608-26802; fax: +49 721 608-26858.

## Sequencing data of the *mcr-1* gene

## The partial sequence of the *mcr-1* gene derived from the 183 bp amplicon. The DNA was extracted from influent and effluent wastewater populations of the WWTPs. Due to the amplicon size it was not possible to upload the sequence to the NCBI BankIt (minimum 200bp).

## Sequence 1: *mcr-1* gene: extracted DNA from wastewater after conventional treatment, partial CDS

## 5´TGGGCCTGCGTATTTTAAGCGATATGATGAAAAGTTTGCCAAATTCACGCCAGTGTGTGAAGGTAATGAGCTTGCCAAGTGCGAACATCAGTCCTTGATCTAATGCTTATGACAATGCCTTGCTTGCCACC3´

## Sequence 2: *mcr-1* gene: extracted DNA from raw wastewater, partial CDS

## 5´TGGGCCTGGCGTATTTTAAGCGATATGATGAAAAGTTTGCCAAATTCACGCCAGTGTGTGAAGGTAATGAGCTTGCCAAGTGCGAACATCAGTCCTTGATCAATGCTTATGACAATGCCTTGCTTGCCACCGGATGCTTTCAT3´

## Calibration curves

### *E. coli* DSM 1103 – *yccT*

Primer:	yccT F (GCATCGTGACCACCTTGA)

yccT R (CAGCGTGGTGGCAAAA)
Annealing temperature: 56 °C

Fragment: 59 bp

Ct

**SI Figure 1**: yccT calibration curve with *E. coli* DSM 1103. The slope S= -3.361 and efficiency E=98.4% was determined with a correlation coefficient of R²=0.994. The mean Ct-value of the 10^-5^ dilution was with 28.26 too high to be within the linear range. Detection limit is equal to 4.01 cell equivalents.

**SI Figure 2:** Melting curve of *yccT* calibration. Consistent melting temperature of 76 °C was detected for most dilutions. Dilution of 10^-5^ to 10^-7^ showed with 75.5 °C a slightly lower melting temperature and slightly lower signal intensity.

### *K. pneumoniae* DSM 30104 – *gltA*

Primer:	gltA F (ACGGCCGAATATGACGAATTC)

gltA R (AGAGTGATCTGCTCATGAA)

Annealing temperature: 56 °C

Fragment: 68 bp

Ct

**SI Figure 3:** *gltA* calibration curve with *K. pneumoniae* DSM 30104. The slope S= -3.387 and efficiency E=97.4% was determined with a correlation coefficient of R²=0.998. Detection limit is equal to 18 cell equivalents.

**SI Figure 4:** Melting curve of *gltA* calibration. Melting temperature varied between 76 °C and 76.5 °C. Signal intensities decrease upon higher dilutions.

### *A. baumannii* DSM 30007 – *secE*

Primer:	secERT-F (GTTGTGGCTTTAGGTTTATTATACG)

secERT-R (AAGTTACTCGACGCAATTCG)

Annealing temperature: 56 °C

Fragment: 94 bp

Ct

**SI Figure 5:** *SecE* calibration curve with A*.baumannii* DSM30007. The slope S= -3.380 and Efficiency E= 97.6% was determined with a correlation coefficient of R²=1. Detection limit is equal to 31 cell equivalents.

**SI Figure 6:** Melting curve of *secE* calibration. Melting temperature was consistent at 77.5 °C. Signal intensities decrease upon higher dilutions.

### *E.coli* NRZ-14408 – *mcr1*

Primer:	mcr1FP (GGGCCTGCGTATTTTAAGCG)

mcr1RP (CATAGGCATTGCTGTGCGTC)

Annealing temperature: 60 °C

Fragment: 183 bp

Ct

**SI Figure 7**: *mcr-1* calibration curve with *E.coli* NRZ-14408. The slope S= -3.380 and efficiency E=97.6% was determined with a correlation coefficient of R²=0.999. Detection limit is equal to 35.2 cell equivalents.

**SI Figure 8**: Melting curve of *mcr-1* calibration. Melting temperature varied between 80 °C and 80.5 °C. Signal intensities decrease upon higher dilutions.

### *E.coli* pNORM – *CTX-M-32*

Primer:	ctxm32-F (CGTCACGCTGTTGTTAGGAA)

ctxm32-R (CGCTCATCAGCACGATAAAG)

Annealing temperature: 60 °C

Fragment: 155 bp

Ct

**SI Figure 9**: *CTX-M-32* calibration curve with *E.coli* pNORM. The slope S= -3.517 and efficiency E=92.5% was determined with a correlation coefficient of R²=1.0. Detection limit is equal to 235 gene copy numbers.

**SI Figure 10**: Melting curve of *CTX-M-32* calibration. Melting temperature was 82.5 °C. Signal intensities decrease upon higher dilutions

### *E.coli* pNORM – *blaTEM*

Primer:	qblaTEM-F (TTCCTGTTTTTGCTCACCCAG)

qblaTEM-R (CTCAAGGATCTTACCGCTGTTG)

Annealing temperature: 60 °C

Fragment: 112 bp

Ct

**SI Figure 11**: *blaTEM* calibration curve with *E.coli* pNORM. The slope S= -3.303 and efficiency E=100.8% was determined with a correlation coefficient of R²=0.999. Detection limit is equal to 80.1 gene copy numbers.

**SI Figure 12**: Melting curve of *blaTEM* calibration. Melting temperature varied between 78 °C and 78.5 °C. Signal intensities decrease upon higher dilutions.

***E.coli* DSM 22664 – *CTX-M***

Primer:	S_CTX-MuF (CGCTTTGCGATGTGCAG)

S_CTX-MuR (ACCGCGATATCGTTGGT)

Annealing temperature: 60 °C

Fragment: 551 bp

Ct

**SI Figure 13**: *CTX-M* calibration curve with *E.coli* DSM 22664. The slope S=-3.504 and efficiency E= 92.9% was determined with a correlation coefficient of R²=1.0. Detection limit is equal to 93.1 gene copy numbers.

**SI Figure 14**: Melting curve of *CTX-M* calibration. Melting temperature was 86 °C. Signal intensities decrease upon higher dilutions.

***E.coli* DH5a – *tetM***

Primer:	S_tetM-F (GGTTTCTCTTGGATACTTAAATCAATCR)

S_tetM-R(CCAACCATAYAATCCTTGTTCRC)

Annealing temperature: 60 °C

Fragment: 88 bp

Ct

**SI Figure 15**: *tetM* calibration curve with *E.coli* DH5a. The slope S= -3.424 and efficiency E= 95.9% was determined with a correlation coefficient of R²=0.998. Detection limit is equal to 3.69 gene copy numbers.

**SI Figure 16**: Melting curve of *tetM* calibration. Melting temperature was 74 °C. Signal intensities decrease upon higher dilutions.

***K. pneumoniae* NRZ01013 – *CMY-2***

Primer:	CMY-2 RT-F (CGTTAATCGCACCATCACC)

CMY-2 RT-R (CGTCTTACTAACCGATCCTAGC)

Annealing temperature: 62 °C

Fragment: 172 bp

Ct

**SI Figure 17**: *CMY-2* calibration curve with *K. pneumoniae*. The slope S= -3.591 and efficiency E= 89.9% was determined with a correlation coefficient of R²=0.998. Detection limit is equal to 70.6 cell equivalents.

**SI Figure 18**: Melting curve of *CMY-2* calibration. Melting temperature was 81 °C. Signal intensities decrease upon higher dilutions.

***S. hyointestinalis* DSM20770/ATCC49169/NCTC12224 – *ermB***

Primer:	ermB-F (TGAATCGAGACTTGAGTGTGCAA)

ermB-R (GGATTCTACAAGCGTACCTT)

Annealing temperature: 60 °C

Fragment: 71 bp

Ct

**SI Figure 19**: *ermB* calibration curve with *S. hyointestinalis*. The slope S= -3.318 and efficiency E= 100.1% was determined with a correlation coefficient of R²=1. Detection limit is equal to 32.1 cell equivalents.

**SI Figure 20**: Melting curve *ermB* calibration. Melting temperature varied between 75 and 75.5 °C. Signal intensities decrease upon higher dilutions.
